# Analyzing the current situation and influencing factors of rural doctors’ intervention in hospice care services in Guangxi

**DOI:** 10.3389/fpubh.2025.1740030

**Published:** 2026-01-14

**Authors:** Shuang Liang, Lang Jiang, Zhao Li

**Affiliations:** 1 Guangxi University of Chinese Medicine, School of Public Health and Management, Nanning, Guangxi, China; 2 School of Public Affairs, Xiamen University, Xiamen, Fujian, China

**Keywords:** Guangxi, hospice care, influencing factors, rural doctors, self-determination theory

## Abstract

**Introduction:**

This study aimed to assess the current status and identify the key determinants of hospice care involvement among rural doctors in Guangxi, China, using Self-Determination Theory (SDT) as a framework.

**Methods:**

A cross-sectional survey was conducted among 368 rural doctors from August to September 2024, with 312 valid responses (84.8% response rate). Data were collected using an online questionnaire assessing hospice care knowledge, willingness to provide care, and SDT-related psychological needs (autonomy, competence, and relatedness). Chi-square tests and ordinal logistic regression were used for analysis.

**Results:**

Respondents demonstrated moderate knowledge of hospice care (8.9% comprehensive) but relatively high willingness (21.7% highly willing). The primary services provided included psychological support (75.0%) and companionship (59.6%). Multivariate analysis revealed that higher knowledge levels were positively associated with greater willingness to participate in research. Paradoxically, lower educational attainment (high school/associate degree vs. bachelor’s degree) was a significantly positive predictor of willingness (OR = 3.07, *p =* 0.002; OR = 2.88, *p =* 0.002). Other key determinants aligned with SDT—professional identity (relatedness) and manageable workload (autonomy)—were positive motivators, whereas perceived unfair medical liability risk and excessive workload were significant barriers.

**Discussion:**

Rural doctors in Guangxi showed a promising willingness to engage in hospice care, but their involvement was significantly constrained by systemic and motivational factors beyond mere knowledge. Interventions grounded in the SDT—such as enhancing competence through targeted training, ensuring autonomy via workload management and legal safeguards, and fostering relatedness through professional support—are essential for leveraging this willingness and improving end-of-life care in rural China.

## 1 Introduction

Ensuring access to dignified end-of-life care is a global public health imperative and key indicator of an equitable healthcare system. In China, rapid population aging has escalated hospice care demand, particularly in rural regions, where the proportion of older adults exceeds that in urban areas (1). While national policy advancements such as the 2024 inclusion of hospice care in medical service pricing guidelines signal growing recognition (2), a profound urban–rural disparity persists, with services and resources predominantly concentrated in cities (3, 4). This gap is particularly concerning because rural residents often face higher barriers in accessing quality end-of-life support (5, 6).

In these resource-constrained settings, village doctors, certified frontline providers in China’s rural health system, serve as indispensable gatekeepers of community health (7, 8). Their unique position within communities makes them potentially key agents for delivering primary-level hospice care (9). However, their capacity to fulfill this role is challenged by systemic constraints, including heavy workloads, limited training opportunities, and inadequate infrastructure (10, 11). Existing research on hospice care in China has largely focused on urban hospitals and licensed medical staff (12), leaving a significant knowledge gap regarding the perceptions, competencies, and motivations of rural doctors, especially in underdeveloped regions, such as the Guangxi Zhuang Autonomous Region.

**Figure 1:**
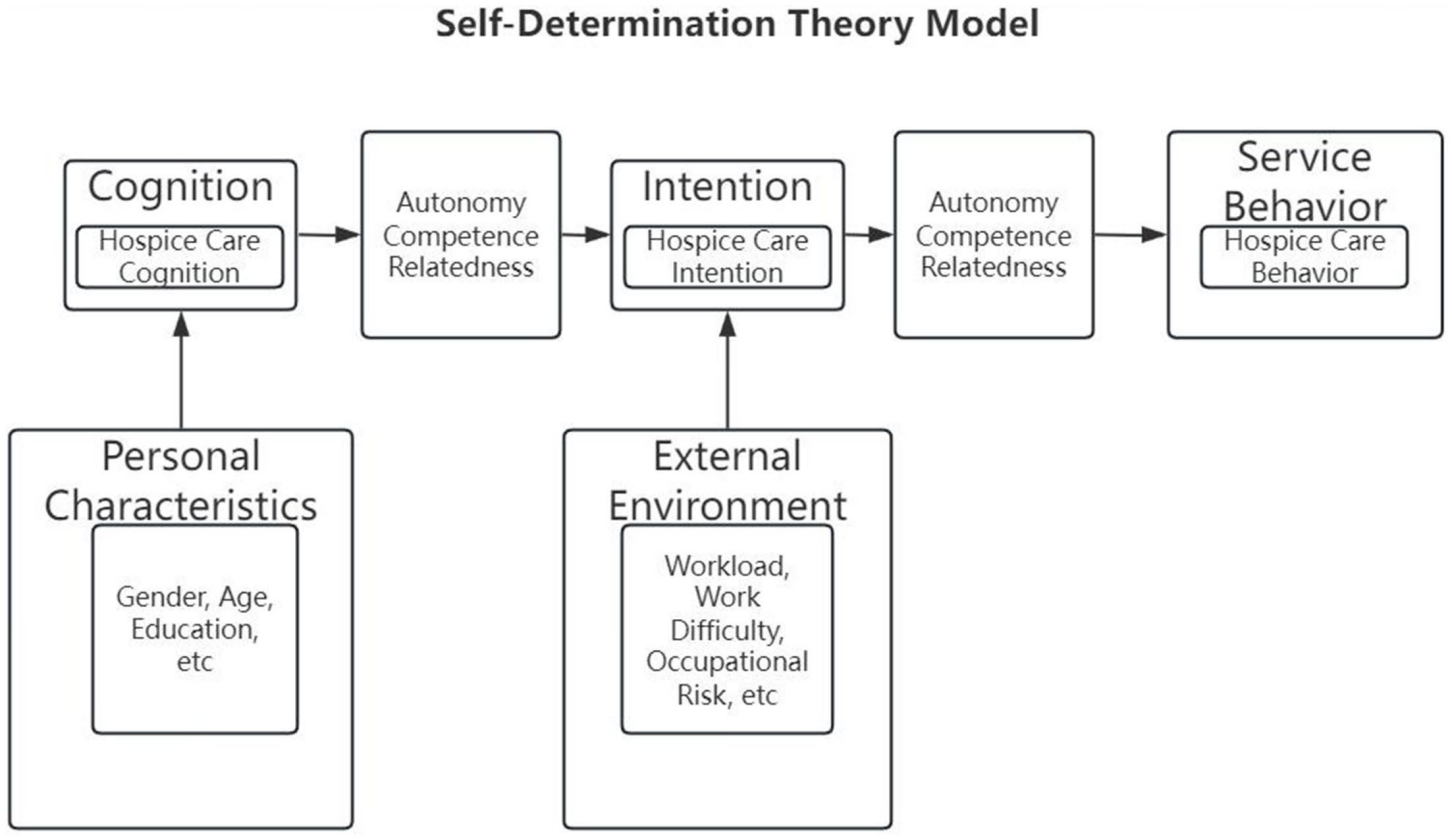
Self-determination theory model.

The Self-Determination Theory (SDT) offers a robust framework (13). The SDT posits that satisfying three basic psychological needs—competence (feeling effective), autonomy (sense of choice), and relatedness (connection to others)—fosters high-quality, self-motivated engagement. Applying this lens, a rural doctor’s willingness to provide hospice care may depend not only on knowledge (competence) but also on factors such as control over workload (autonomy) and supportive professional relationships (relatedness). Although the SDT has been applied in healthcare, its use in investigating rural hospice care engagement remains novel (see Figure 1).

This study aimed to bridge this critical gap by investigating the status and determinants of hospice care involvement among rural doctors in Guangxi, China. The specific objectives were as follows: (1) to assess their level of knowledge and willingness regarding hospice care; (2) to identify the sociodemographic, occupational, and psychosocial factors (informed by the SDT) associated with these outcomes; and (3) to provide evidence-based recommendations for policy and practice. We hypothesized that higher knowledge levels and factors supporting psychological needs satisfaction (e.g., professional identity and fair workload) would be positively associated with a greater willingness to provide care, whereas perceived barriers (e.g., high medical liability risk and low-income satisfaction) would exhibit negative associations.

## 2 Method

### 2.1 Study design and reporting guidelines

A cross-sectional survey was conducted among rural doctors in the Guangxi Zhuang Autonomous Region between August and September 2024. This study was designed in accordance with the Strengthening the Reporting of Observational Studies in Epidemiology (STROBE) guidelines for cross-sectional studies.

### 2.2 Participants and sampling

A two-stage stratified sampling method was used. First, 11 towns were randomly selected from each of the 14 prefecture-level cities in Guangxi for a total of 154 towns. Second, a list of eligible rural doctors was created for each town. From this list, two to three doctors were selected via simple random sampling (two if fewer than five eligible doctors were available, and three if five or more were available).

The sample size was calculated *a priori* using G*Power 3.1 software. The parameters were set as follows: *α* = 0.05, β = 0.2 (power = 80%), effect size *f* = 0.05 (small effect), and 12 predictor variables. A minimum sample size of 286 participants was required for this study. Accounting for an estimated 20% non-response rate, 368 questionnaires were distributed to participants.

### 2.3 Inclusion and exclusion criteria

The inclusion criteria were as follows: (1) registered rural doctors holding a valid practice certificate, (2) working in a village clinic within one of the 154 selected towns, (3) having contact with terminally ill patients (e.g., those with advanced chronic diseases) in daily practice, and (4) providing voluntary informed consent.

The exclusion criteria were as follows: (1) administrative or logistical staff in village clinics not directly involved in clinical services, (2) those on long-term leave (>3 months) during the survey period, and (3) those with severe physical or mental illness that impaired their ability to complete the questionnaire.

### 2.4 Data collection and ethical considerations

Data were collected using an online structured questionnaire distributed through the Wenjuanxing platform. Before distribution, the research team coordinated with local health administrations at the prefectural and township levels to facilitate the survey. Township health centers sent the questionnaire link to eligible rural doctors via WeChat.

A total of 368 questionnaires were submitted. All responses were subjected to rigorous two-step validation. First, to prevent duplicate responses, the Wenjuanxing platform was configured to allow only one submission per unique WeChat ID and IP address. Second, two trained research assistants independently performed a manual review of all 368 records included in the study. Invalid questionnaires were excluded based on the following pre-defined criteria: (1) missing data for key demographic variables (e.g., age, certification) or the core dependent variables (knowledge or willingness); (2) logical inconsistencies (e.g., selecting “no knowledge of hospice care” but later detailing specific services provided); or (3) evidence of random or inattentive responding, such as identical answers across all matrix questions (straight-lining) or implausibly short completion times (< one-third of the median time). Following this screening, 56 questionnaires were excluded, resulting in 312 valid questionnaires for analysis, a valid response rate of 84.8%.

The study protocol was reviewed and approved by the Ethics Committee of Guangxi University of Chinese Medicine (Approval No.: GXTCMU-EC KS20251222-04). All the procedures adhered to the principles of the Declaration of Helsinki. The first page of the online questionnaire presented a detailed electronic informed consent form outlining the purpose of the study, procedures, potential risks and benefits, data confidentiality, and the voluntary nature of participation. The participants were required to provide consent before proceeding. No direct financial incentives were provided; however, completion of the survey was acknowledged as a continuing educational activity by the supporting local health bureaus. De-identified data supporting these findings may be made available from the corresponding author upon request.

### 2.5 Questionnaire development and measures

The questionnaire was developed through a literature review and expert interviews and comprised 56 items across four domains: (1) sociodemographic and occupational characteristics, (2) hospice care knowledge, (3) hospice care willingness, and (4) psychological needs related to the SDT (autonomy, competence, and relatedness).

Hospice care knowledge was assessed using participants’ self-rated familiarity. The willingness to provide hospice care was measured using a Likert scale. The key independent variables included education level, professional identity, satisfaction with medical and public health workloads, perceived fairness of workload and medical liability risk, perceived job difficulty, and quality of professional relationships.

The formal questionnaire demonstrated good internal consistency, with a Cronbach’s *α* coefficient of 0.89. A parallel analysis confirmed a five-factor structure that accounted for 65% of the total variance.

### 2.6 Statistical analysis

Data were cleaned using Microsoft Excel 2019 and analyzed using SPSS version 25.0. Descriptive statistics were calculated for all variables. Chi-square tests were performed for the univariate analysis to identify factors associated with hospice care knowledge and willingness to participate.

Ordered logistic regression was used for multivariate analysis, with knowledge and willingness as ordinal dependent variables. The parallel lines test confirmed that the proportional odds assumption was met (χ^2^ = 24.354, df = 23, *p =* 0.384), validating the use of this model. The model fit was assessed using the chi-square model and goodness-of-fit statistics.

Strategies to Address Potential Biases: To assess non-response bias, we compared early respondents (first 75%) with late respondents (last 25%) on key demographics (age, sex, and education) using chi-square tests; no significant differences were found (all *p* > 0.05). Harman’s single-factor test was used to evaluate the risk of common method bias. The unrotated factor solution revealed that the first factor accounted for 28.7% of the variance, which was below the 50% threshold, suggesting that common method bias was not a major concern. Missing data were minimal (<2% for any variable) and were assumed to be missing completely at random (MCAR). Cases with missing dependent variables in the regression models were excluded.

## 3 Results

### 3.1 Demographic and professional characteristics of participants

Of the 368 questionnaires distributed, 312 were retained for analysis, yielding a valid response rate of 84.8%. The demographic and occupational profiles of the respondents are summarized in Table 1. The sample comprised more male (57.7%) than female (42.3%) village doctors, with the majority (63.1%) aged between 40 and 60 years. In terms of educational attainment, 36.2% held an associate’s degree, 28.8% completed high school or technical secondary school, and 28.8% had a bachelor’s degree or higher. Most worked in standardized clinics (81.7%) operating under the national essential drug system (91.6%). A significant proportion of physicians combined medical practice with farming, with only 28.8% practicing medicine full-time. The most common monthly income brackets were RMB 3,001–4,000 (30.4%) and RMB 2,001–3,000 (29.3%).

**Table 1:** Demographic and occupational characteristics of participating rural doctors (*N* = 312).

Characteristic	Category	*n*	%
Gender	Male	178	57.1
	Female	134	42.9
Age (years)	< 30	35	42.9
	30–49	174	56.7
	50–69	86	28.0
	≥ 70	12	3.9
Marital status	Unmarried	40	12.8
	Married	259	83.0
	Divorced	7	2.2
	Widowed	6	1.9
Education	Primary school or below	5	1.6
	Junior high school	17	5.4
	High school/technical secondary school	87	27.9
	Associate degree	113	36.2
	Bachelor’s degree or above	90	28.8
Monthly income (RMB)	≤ 2,000	79	25.3
	2,001–3,000	93	29.8
	3,001–4,000	89	28.5
	≥ 4,001	51	16.3
Primary occupation	Full-time medical practice	90	28.8
	Mainly medical practice, supplemented by farming	100	32.1
	Equal time on medical practice and farming	98	31.4
	Mainly farming, supplemented by medical practice	19	6.1
	Other	5	1.6
Professional certification	Assistant medical practitioner	51	16.3
	Medical practitioner (physician)	33	10.6
	Rural doctor practice certificate	210	67.3
	Other	18	5.8

Percentages were calculated based on the total number of valid responses for each characteristic. Age data were missing for five participants.

### 3.2 Current status of hospice care knowledge, willingness, and practice

As presented in Table 2, respondents exhibited moderate overall knowledge of hospice care. Only 8.9% reported a comprehensive understanding, 41.3% had a relatively good understanding, and 45.2% had only general familiarity. Participants reported a generally positive willingness to provide rural hospice care: 21.7% were highly willing, 41.3% moderately willing, and 32.0% neutral. The primary services currently provided were psychological care (75.0%), companionship, and comfort (59.6%). The most-cited motivations for willingness to participate were professional dedication (53.3%) and personal compassion (32.5%), whereas the main reasons for unwillingness were perceived insufficient ability (40.0%) and an overly heavy workload (33.3%).

**Table 2:** Current status of hospice care knowledge, willingness, and practice among rural doctors (*N* = 312).

Classification	Specific content	*n*	%
Understanding of hospice care services	Very well understand	28	9.0
	Relatively understanding	103	33.0
	General	141	45.2
	Less understanding	30	9.6
	Very unclear	10	3.2
Willingness to receive hospice care services	Very willing	68	21.7
	More willing	129	41.3
	General	100	32.0
	Relatively unwilling	12	3.8
	Very reluctant	3	0.9
Reasons for being willing to participate in hospice care services	Personal feelings	64	32.5
	Work tasks	27	13.7
	Dedication and devotion	105	53.3
	Others	1	0.5
Reasons for unwillingness to participate in hospice care services	Insufficient ability	6	40.0
	The work tasks are heavy, and there is no time.	5	33.3
	There are no such indicators in the performance evaluation.	3	20.0
	Others	1	6.7
Available hospice care services	Prescribe painkillers	209	67.0
	Accompany and comfort	186	59.6
	Daytime care	130	42.7
	Provide psychological care	234	75.0
	Others	7	2.2

### 3.3 Univariate analysis of factors associated with knowledge and willingness

Table 3 provides a detailed coding scheme (variable assignment) for all independent and dependent variables included in the univariate and subsequent multivariate analyses. This clarifies how the Likert scale and continuous variables were categorized in the analysis.

**Table 3:** Variable assignment for factors influencing rural doctors’ awareness and willingness regarding hospice care services.

Variable	Assignment
Gender	1 = Male, 2 = Female
Age	1 = 0~<29, 2= > 30~49, 3= > 50~69, 4 = ≧70
Years of practice	1 = 0~<8, 2= > 9~16, 3= > 17~24, 4 = ≧25
Public health workload	1 = Low (including very low and somewhat low), 2 = Moderate, 3 = High (including somewhat high and very high)
Satisfaction with medical workload	1 = Dissatisfied (including very dissatisfied and somewhat dissatisfied), 2 = Moderate, 3 = Satisfied (including somewhat satisfied and very satisfied)
Perceived fairness of workload distribution	1 = Unfair (including very unfair and somewhat unfair), 2 = Moderate, 3 = Fair (including somewhat fair and very fair)
Perceived fairness of medical liability risk	1 = Unfair (including very unfair and somewhat unfair), 2 = Moderate, 3 = Fair (including somewhat fair and very fair)
Satisfaction with average income	1 = Dissatisfied (including very dissatisfied and somewhat dissatisfied), 2 = Moderate, 3 = Satisfied (including somewhat satisfied and very satisfied)
Perceived job difficulty	1 = Disagree (including strongly disagree and somewhat disagree), 2 = Moderate, 3 = Agree (including somewhat agree and strongly agree)
Perceived job pressure	1 = Disagree (including strongly disagree and somewhat disagree), 2 = Moderate, 3 = Agree (including somewhat agree and strongly agree)
Perceived Job complexity	1 = Disagree (including strongly disagree and somewhat disagree), 2 = Moderate, 3 = Agree (including somewhat agree and strongly agree)
Adequacy of infrastructure	1 = Disagree (including strongly disagree and somewhat disagree), 2 = Moderate, 3 = Agree (including somewhat agree and strongly agree)
Relationship with leadership	1 = Poor (including very poor and somewhat poor), 2 = Moderate, 3 = Good (including somewhat good and very good)
Relationship with physicians	1 = Poor (including very poor and somewhat poor), 2 = Moderate, 3 = Good (including somewhat good and very good)
Relationship with colleagues	1 = Poor (including very poor and somewhat poor), 2 = Moderate, 3 = Good (including somewhat good and very good)
Relationship with patients	1 = Poor (including very poor and somewhat poor), 2 = Moderate, 3 = Good (including somewhat good and very good)
Availability of upward feedback channels	1 = Disagree (including strongly disagree and somewhat disagree), 2 = Moderate, 3 = Agree (including somewhat agree and strongly agree)
Timely response to raised issues	1 = Disagree (including strongly disagree and somewhat disagree), 2 = Moderate, 3 = Agree (including somewhat agree and strongly agree)
Perceived fairness of policy implementation	1 = Disagree (including strongly disagree and somewhat disagree), 2 = Moderate, 3 = Agree (including somewhat agree and strongly agree)
Awareness of hospice care services	1 = Knowledgeable (including very knowledgeable and somewhat knowledgeable), 2 = Moderate, 3 = Not knowledgeable (including somewhat unknowledgeable and very unknowledgeable)
Willingness to provide hospice care services	1 = Willing (including very willing and somewhat willing), 2 = Moderate, 3 = Unwilling (including somewhat unwilling and very unwilling)

Univariate analyses using chi-square tests identified several factors that were significantly associated with hospice care knowledge and willingness to participate. The results are detailed in Tables 4, 5, where statistically significant *p*-values (*p* < 0.05) are presented in bold.

**Table 4:** Univariate analysis of rural doctors’ awareness of hospice care services.

Item	Awareness of hospice care services			χ^2^	*P-value*
	Knowledgeable	Moderate	Not		
Gender				4.762	0.092
Male	66	85	27		
Female	65	56	13		
Age				**14.978**	**0.020**
0 ~ <29	17	10	4		
>30~49	68	89	17		
>50~69	36	29	15		
≧70	4	17	15		
Years of practice				5.499	0.482
0~<8	44	39	7		
>9~16	30	40	11		
>17~24	25	30	12		
≧25	30	33	10		
Public health workload				4.753	0.314
Low	16	24	5		
Moderate	66	78	25		
High	49	39	40		
Satisfaction with medical workload				**15.711**	**0.003**
Dissatisfied	17	10	3		
Moderate	37	68	22		
Satisfied	77	63	15		
Perceived fairness of workload				**14.881**	**0.005**
Unfair	15	8	3		
Moderate	35	66	20		
Fair	81	67	17		
Perceived fairness of medical liability risk				**20.848**	**<0.001**
Unfair	15	18	13		
Moderate	48	74	16		
Fair	68	49	11		
Satisfaction with average income				6.711	0.152
Dissatisfied	23	21	10		
Moderate	44	64	17		
Satisfied	64	56	13		
Perceived job difficulty				**13.333**	**0.01**
Disagree	22	23	12		
Neutral	53	80	18		
Agree	56	38	10		
Perceived job pressure				8.111	0.088
Disagree	25	23	14		
Neutral	53	67	15		
Agree	53	51	11		
Adequacy of infrastructure				**16.485**	**0.002**
Disagree	19	33	14		
Neutral	48	59	19		
Agree	64	49	7		
Relationship with leadership				**23.145**	**<0.001**
Poor	7	7	4		
Neutral	17	52	13		
Good	107	82	23		
Relationship with physicians				**20.750**	**<0.001**
Poor	7	9	3		
Neutral	19	52	15		
Good	105	80	22		
Relationship with colleagues				**15.137**	**0.004**
Poor	7	6	4		
Neutral	12	31	12		
Good	112	104	24		
Relationship with patients				**12.149**	**0.016**
Poor	4	7	2		
Neutral	15	37	11		
Good	112	97	27		
Availability of upward feedback channels				**11.627**	**0.020**
Disagree	6	13	7		
Neutral	35	42	16		
Agree	90	86	17		
Timely response to raised issues				**16.075**	**0.003**
Disagree	7	13	9		
Neutral	39	54	16		
Agree	85	74	15		
Perceived fairness of policy implementation				**15.183**	**0.004**
Unfair	6	15	8		
Moderate	38	50	17		
Fair	87	76	15		

**Table 5:** Univariate analysis of rural doctors’ willingness to provide hospice care services.

Item	Willingness to Provide hospice care Services			χ^2^	*P-value*
	Willing	Neutral	Unwilling		
Gender				5.951	0.051
Male	103	67	8		
Female	94	33	7		
Age				3.321	0.768
0~<29	22	8	1		
>30~49	112	54	8		
>50~69	48	28	4		
≧70	3	3	1		
Years of practice				4.456	0.615
0~<8	63	24	3		
>9~16	52	25	5		
>17~24	40	22	4		
≧25	40	29	4		
Public health workload				4.236	0.375
Low	29	15	1		
Moderate	111	52	6		
High	57	33	8		
Satisfaction with medical workload				**20.123**	**<0.001**
Dissatisfied	17	10	3		
Moderate	65	57	5		
Satisfied	115	33	7		
Perceived fairness of workload				**22.413**	**<0.001**
Unfair	14	10	2		
Moderate	59	55	7		
Fair	124	35	6		
Perceived fairness of medical liability risk				**21.033**	**<0.001**
Unfair	21	21	4		
Moderate	78	55	5		
Fair	98	24	6		
Satisfaction with average income				**18.798**	**0.001**
Dissatisfied	23	26	5		
Moderate	77	45	3		
Satisfied	97	29	7		
Perceived job difficulty				**10.199**	**0.037**
Disagree	26	28	3		
Neutral	102	43	6		
Agree	69	29	6		
Perceived job pressure				3.551	0.470
Disagree	34	24	4		
Neutral	84	45	6		
Agree	79	31	5		
Perceived job complexity				7.888	0.096
Disagree	28	27	3		
Neutral	86	41	7		
Agree	83	32	5		
Adequacy of infrastructure				**10.803**	**0.029**
Disagree	32	31	3		
Neutral	79	40	7		
Agree	86	29	5		
Relationship with leadership				**11.890**	**0.018**
Poor	9	7	2		
Neutral	42	37	3		
Good	146	56	10		
Relationship with physicians				3.645	0.456
Poor	10	7	2		
Neutral	50	32	4		
Good	137	61	9		
Relationship with colleagues				6.860	0.143
Poor	7	8	2		
Neutral	31	20	4		
Good	159	72	9		
Relationship with patients				2.160	0.706
Poor	6	6	1		
Neutral	38	22	3		
Good	153	72	11		
Availability of upward feedback channels				**15.205**	**0.004**
Disagree	10	15	1		
Neutral	51	36	6		
Agree	136	49	8		
Timely response to raised issues				**9.907**	**0.042**
Disagree	13	14	2		
Neutral	62	40	7		
Agree	122	46	6		
Perceived fairness of policy implementation					
Unfair	13	13	3		
Moderate	62	38	5		
Fair	122	49	7		
Awareness of hospice care services				**74.621**	**<0.001**
Knowledgeable	115	72	10		
Moderate	15	62	23		
Not knowledgeable	1	7	7		

Factors associated with knowledge (Table 4): significant factors (*p* < 0.05) included age, satisfaction with medical workload, perceived fairness of medical liability risk, and availability of upward feedback channels. Factors showing highly significant associations (*p* < 0.001) included education level, relationship with leadership, and perceived fairness of policy implementation.

Factors associated with willingness (Table 5): significant factors (*p* < 0.05) included satisfaction with the medical workload, perceived fairness of workload distribution, medical liability risk, and infrastructure adequacy. Highly significant associations (*p* < 0.001) were found between average income satisfaction and the level of hospice care knowledge.

### 3.4 Multivariate analysis: determinants of knowledge and willingness

The parallel lines test confirmed that the proportional odds assumption was met (χ^2^ = 24.354, *p =* 0.384), thus validating the use of ordinal logistic regression models (Table 6).

**Table 6:** Parallel lines test results.

Model	−2 Log likelihood	Chi-square	df	Significance
Null hypothesis	473.315	–	–	–
Conventional	448.961	24.354	23	0.384

#### 3.4.1 Determinants of hospice care knowledge

The multivariate model for knowledge demonstrated a good fit (final model χ^2^ = 81.949, *p* < 0.001). As shown in Table 7, after controlling for other variables, several factors remained significant independent predictors. Doctors under 29 years of age had significantly lower odds of having more knowledge than those aged 70 years and older (OR = 0.11, *p =* 0.019). Dissatisfaction with medical workload was also a negative predictor. Conversely, perceiving medical liability risk as unfair, having access to upward feedback channels, and perceiving policy implementation as fair were associated with significantly higher odds of possessing knowledge. Furthermore, dissatisfaction with professional identity was a strong negative predictor (OR = 0.20, *p =* 0.011).

**Table 7:** Ordinal logistic regression analysis of rural doctors’ awareness of hospice care services.

Item	B	SE	Wald χ^2^ value	*P-value*	OR(95%CI)
Gender					
Male	0.454	0.261	3.030	0.082	1.575(−0.057, 0.965)
Female	0^*^				
Age					
0~<29	−2.209	0.941	5.507	**0.019**	**0.110(−4.054, −0.364)**
>30~49	−1.354	0.825	2.690	0.101	0.258(−2.971, 0.264)
>50~69	−1.574	0.836	3.543	0.060	0.207(−3.214, 0.065)
≧70	0				
Satisfaction with medical (Ref: ≥70) workload					
Dissatisfied	−1.298	0.545	5.669	**0.017**	**0.273(−2.366, −0.229)**
Moderate	0.541	0.298	3.294	0.070	1.718(−0.043, 1.126)
Satisfied	0				
Perceived fairness of medical liability risk					
Unfair	1.009	0.435	5.368	**0.021**	**2.743(0.155, 1.862)**
Moderate	0.399	0.303	1.731	0.188	1.490(−0.195, 0.994)
Fair	0				
Perceived job difficulty					
Disagree	0.830	0.376	4.874	**0.027**	**2.293(0.093, 1.567)**
Neutral	0.286	0.295	0.939	0.333	1.331(−0.293, 0.865)
Agree	0				
Availability of upward					
Disagree	1.375	0.562	5.994	**0.014**	**3.955(0.274, 2.476)**
Neutral	0.079	0.350	0.052	0.820	1.082(−0.606, 0.765)
Agree	0				
Perceived fairness of policy					
Disagree	1.247	0.537	5.394	**0.020**	**3.480(0.195, 2.300)**
Neutral	0.401	0.333	1.446	0.229	1.493(−0.253, 1.055)
Agree	0				
Professional identity					
Disagree	−1.608	0.634	6.423	**0.011**	**0.200(−2.851, −0.364)**
Neutral	−0.056	0.331	0.029	0.866	0.946(−0.704, 0.593)
Agree	0				
Professional qualification held (Ref: ≥70)					
Assistant practicing physician	−1.293	0.648	3.981	**0.046**	**0.274(−2.564, −0.023)**
Practicing physician	−0.652	0.665	0.961	0.327	0.521(−1.956, 0.652)
Rural doctor practice certificate	−0.462	0.600	0.592	0.442	0.630(−1.638, 0.715)
None	0				

*The last category of each variable was used as the reference group. Bold values indicate that the factor is significantly associated with the dependent variable (*P*<0.05).

#### 3.4.2 Determinants of willingness to provide hospice care

The model for willingness also showed a good fit (final model χ^2^ = 95.514, *p* < 0.001). Table 8 presents the results are presented in Table 8. A key finding was that educational level was negatively associated with the willingness to participate in the adjusted model. Compared with doctors with a bachelor’s degree or higher, those with a high school/technical school (OR = 3.07, *p* = 0.002) or associate’s degree (OR = 2.88, *p* = 0.002) were more willing to provide care. A neutral (versus satisfied) attitude towards the medical workload and professional identity, as well as a disagreement that work was difficult, were also associated with greater willingness.

**Table 8:** Ordinal logistic regression analysis of rural doctors’ willingness to provide hospice care services.

Item	B	SE	Wald χ^2^value	*P-value*	OR(95%CI)
Gender					
Male	0.377	0.261	2.082	0.149	1.458(−0.135, 0.888)
Female	0^*^				
Education level					
Primary school or below	1.793	0.949	3.571	0.059	6.007(−0.067, 3.652)
Junior high school	1.095	0.591	3.432	0.064	2.989(−0.064, 2.253)
High school/technical secondary school	1.122	0.365	9.463	**0.002**	**3.071(0.407, 1.837)**
Associate degree	1.059	0.345	9.426	**0.002**	**2.883(0.383, 1.736)**
Bachelor’s degree or above	0				
Satisfaction with medical workload					
Dissatisfied	0.649	0.425	2.336	0.126	1.914(−0.183, 1.481)
Moderate	0.849	0.272	9.719	**0.002**	**2.337(0.315, 1.382)**
Satisfied	0				
Perceived job difficulty					
Disagree	0.838	0.409	4.208	**0.040**	**2.312(0.037, 1.639)**
Neutral	−0.120	0.332	0.131	0.717	0.887(−0.770, 0.530)
Agree	0				
Professional identity					
Disagree	0.841	0.508	2.743	0.098	2.319(−0.154, 1.837)
Neutral	0.956	0.298	10.304	**0.001**	**2.601(0.372, 1.540)**
Agree	0				
Perceived fairness of medical liability risk					
Unfair	−0.419	0.417	1.010	0.315	0.658(−1.237, 0.398)
Moderate	−0.207	0.331	0.389	0.533	0.813(−0.856, 0.443)
Fair	0				

*The last category of each variable was set as the reference group: OR, Exp (B). Bold values indicate that the factor is significantly associated with the dependent variable (*P*<0.05).

The ordinal logistic regression model for willingness (Table 8) identified several independent predictors after adjustment. Notably, a lower educational level (high school/technical school or associate’s degree, compared to a bachelor’s degree or above) was significantly associated with greater willingness to provide hospice care (OR = 3.07, *p =* 0.002 and OR = 2.88, *p =* 0.002, respectively). Additionally, neutral (versus satisfied) attitudes towards medical workload and professional identity were positive predictors of willingness.

## 4 Discussion

### 4.1 Principal findings and integration with research framework

This study provides novel insights into hospice care engagement among village doctors in the underdeveloped regions of China. Our findings revealed a complex interplay between knowledge, motivation, and context. In response to our research questions, we found that rural doctors in Guangxi possessed a moderate level of knowledge, but a relatively high willingness to provide hospice care (RQ1). This willingness is paradoxically higher among those with lower formal education and is significantly shaped by a constellation of occupational, psychological, and systemic factors (RQ2), as follows: The results largely support our hypotheses derived from SDT. Factors indicative of psychological need satisfaction such as professional identity (relatedness) and manageable workload (autonomy) were positively associated with engagement, whereas systemic barriers such as perceived unfair liability risk negatively impacted engagement (H2). Crucially, a strong positive correlation between knowledge level and willingness was confirmed (H1), but this relationship was moderated by the work context, underscoring the SDT’s relevance in this setting.

### 4.2 Interpretation of key determinants and theoretical implications

#### 4.2.1 The knowledge-willingness nexus and the role of education

The positive correlation between knowledge and willingness aligns with studies among urban healthcare professionals (14, 15), confirming that understanding fosters intent. However, the negative association between higher education (bachelor’s degree) and willingness in multivariate analysis is a striking and counterintuitive finding. This contrasts with reports from urban settings, where education often predicts greater palliative care engagement (16). An SDT-informed explanation posits that highly educated doctors in resource-poor villages may experience profound deficits in perceived competence. Their training may have equipped them with ideals that were unattainable in a setting lacking essential medications and support (10), leading to frustration and reduced motivation to engage in complex and emotionally demanding hospice care. Conversely, doctors with secondary- or associate-level education may perceive their skills as better matched to the available resources, fostering a greater sense of efficacy and willingness to work.

#### 4.2.2 Professional identity as a core motivator

Professional identity emerged as one of the strongest positive factors. Most willing participants cited “dedication” and “conscience” as their primary motivators. This aligns with the SDT’s relatedness needs, reflecting a deep-seated connection to their role as community guardians (17, 18). For many village doctors, providing companionship at the end of life is a fundamental part of their identity, offering an intrinsic fulfillment that transcends material incentives. This finding highlights a vital asset upon which interventions can be built: existing humanistic commitment within the rural medical workforce.

#### 4.2.3 Systemic barriers: workload, liability, and economic constraints

The negative impact of high medical workload satisfaction on willingness underscores critical system failure. This suggests that doctors who are already saturated with clinical and public health duties perceive hospice care as an unsustainable addition that erodes their sense of autonomy (19, 20). Furthermore, the perception of unfair medical liability risk was a significant barrier, consistent with concerns documented in other settings (21, 22). The fear of disputes is a powerful deterrent to the absence of clear legal safeguards or risk-sharing mechanisms.

Economic constraints underlie several of these barriers. The reported low income levels, coupled with the lack of specific funding for hospice care, create a fundamental mismatch. The additional time required for effective palliative support represents an uncompensated opportunity cost, making sustained engagement economically impractical for many and creating a bottleneck in rural healthcare globally (5).

### 4.3 Recommendations for policy and practice

A multilevel strategy is required to translate the identified determinants into actionable policies. The following recommendations are made to systematically enhance the competence, autonomy, and relatedness of rural doctors, thereby fostering a sustainable environment for hospice care integration.

#### 4.3.1 Establish a tiered, competency-based training system

A foundational step is to address this critical knowledge gap by developing a mandatory, standardized, yet locally adaptable, training curriculum. This program should be integrated into the continuing education framework for rural doctors by leveraging platforms such as the Guangxi Primary Health Care Talent Enhancement Project. Training must move beyond theory to focus on pragmatic skills, such as symptom management with limited medications (10), effective communication with families in a rural context, and self-care strategies to prevent burnout. Formal certification from such training validates hospice care as a core professional competency, directly strengthening the professional identity, which is a key motivator, and enhancing their sense of competence (13, 23).

#### 4.3.2 Create a supportive service network and resource guarantee mechanism

The systemic barriers of perceived high liability risk and an overwhelming workload must be addressed through structural support. This requires clearly defining hospice care services within rural doctors’ contractual responsibilities and establishing formalized referral pathways for specialist support at townships and county hospitals. This safety net reduces the burden of sole responsibility and mitigates the fear of unfair medical liability risk (21, 22). Concurrently, policy reform must ensure the availability of essential palliative drugs (e.g., opioids) in village clinic formularies (10). Furthermore, promoting interdisciplinary teamwork by connecting rural doctors with nurses, public health workers, and potentially medical social workers (24) distributes the clinical and emotional load, builds professional relatedness, and counteracts the autonomy-eroding effect of isolated practice (13).

#### 4.3.3 Implement integrated economic incentives and legal safeguards

Sustainable engagement requires economic and legal incentives. A multicomponent compensation model that links financial incentives to service quality, duration, and family satisfaction metrics should be introduced. This directly addresses the economic constraints identified in our study and offsets the opportunity costs of providing time-intensive care (5). Simultaneously, legal and risk-protection frameworks must be strengthened. This includes developing provincial practice guidelines to standardize care and explicitly incorporate hospice care services into medical liability insurance schemes (21, 25). These measures provide external security and perceived fairness necessary for autonomous and confident practices.

#### 4.3.4 Cultivate a receptive socio-cultural environment through education

Long-term success depends on changing the community context in which care is provided. Public awareness campaigns are needed to reframe perceptions of hospice care and address deep-seated cultural norms surrounding death and filial piety (26, 27). This reduces community resistance and aligns family expectations with palliative care goals. Simultaneously, establishing peer support networks for rural doctors is crucial for sustaining their psychological well-being, reinforcing their sense of shared purpose (relatedness), and building resilience against the emotional challenges inherent in end-of-life care (13).

### 4.4 Limitations

This study had several limitations. First, its cross-sectional design limits causal inferences regarding the relationship between motivational factors and behavioral intentions. Second, although we aimed for a broad sample, the online survey format may have excluded older doctors who were less familiar with digital tools, potentially introducing selection bias. Third, despite checking for common method bias, our reliance on self-reported data is vulnerable to social desirability bias. Fourth, although guided by the Self-Determination Theory, measurement constraints prevented us from conducting a formal mediation analysis to validate the proposed psychological pathways statistically. Finally, the geographic focus on Guangxi Province may have affected the generalizability of our findings to other regions of China with different socioeconomic landscapes.

These limitations indicate clear opportunities for future research. To establish causality, longitudinal or experimental studies are required to trace how the SDT constructs (autonomy, competence, and relatedness) influence long-term engagement in hospice care. To deepen contextual insights, mixed-method approaches combining surveys with qualitative interviews can richly capture the lived experiences and emotional challenges of rural doctors. Intervention studies are crucial for testing the effectiveness of training programs or new service models grounded in SDT-principles to bridge theory and practice. Finally, to assess broader relevance, comparative multiregional studies within China would help determine the generalizability of our findings and identify region-specific moderating factors in future studies.

## 5 Conclusion

This study moves beyond documenting the knowledge gap to unravel the motivational anatomy of rural doctors’ engagement with hospice care. This demonstrates that willingness is not merely a function of individual compassion but is critically mediated by the work context, its structural fairness, resource availability, and alignment with professional identity. The application of Self-Determination Theory provides a powerful lens for understanding these dynamics. Therefore, enhancing rural hospice care in China requires a paradigm shift from asking for overburdened individuals to redesigning systems that actively support autonomy, competence, and relatedness. By addressing the economic, legal, and educational bottlenecks, policymakers can translate the latent willingness of Guangxi village doctors into sustainable, high-quality end-of-life care, serving as a model for other underdeveloped regions worldwide.

## Data Availability

The original contributions presented in the study are included in the article/supplementary material, further inquiries can be directed to the corresponding author.
